# Endobronchial ultrasound elastography strain ratio for mediastinal lymph node diagnosis

**DOI:** 10.1515/raon-2015-0020

**Published:** 2015-11-27

**Authors:** Ales Rozman, Mateja Marc Malovrh, Katja Adamic, Tjasa Subic, Viljem Kovac, Matjaz Flezar

**Affiliations:** 1 University Clinic of Pulmonary and Allergic Diseases Golnik, Golnik, Slovenia; 2 Institute of Oncology Ljubljana, Ljubljana, Slovenia

**Keywords:** cancer staging, elastography, endobronchial ultrasound, lung cancer, needle biopsy

## Abstract

**Background:**

Ultrasound elastography is an imaging procedure that can assess the biomechanical characteristics of different tissues. The aim of this study was to define the diagnostic value of the endobronchial ultrasound (EBUS) elastography strain ratio of mediastinal lymph nodes in patients with a suspicion of lung cancer. The diagnostic values of the strain ratios were compared with the EBUS brightness mode (B-mode) features of selected mediastinal lymph nodes and with their cytological diagnoses.

**Patients and methods:**

This prospective, single-centre study enrolled patients with an indication for biopsy and mediastinal staging after a non-invasive diagnostic workup of a lung tumour. EBUS with standard B-mode evaluation and elastography with strain ratio measurement were performed before endobronchial ultrasound-guided transbronchial needle aspiration (EBUS-TBNA).

**Results:**

Thirty-three patients with 80 suspicious mediastinal lymph nodes were included. Malignant infiltration was confirmed in 34 (42.5%) lymph nodes. The area under the receiver operating characteristic curve for the strain ratio was 0.87 (*p* < 0.0001). At a strain ratio ≥ 8, the accuracy for malignancy prediction was 86.25% (sensitivity 88.24%, specificity 84.78%, positive predictive value [PPV] 81.08%, negative predictive value [NPV] 90.70%). The strain ratio is more accurate than conventional B-mode EBUS modalities for differentiating between malignant and benign lymph nodes.

**Conclusions:**

EBUS-guided elastography with strain ratio assessment can distinguish malignant from benign mediastinal lymph nodes with greater accuracy than conventional EBUS modalities. This new method may reduce the number of mediastinal EBUS-TBNAs and thus reduce the invasiveness and expense of mediastinal staging in patients with non-small lung cancer (NSCLC).

## Introduction

Mediastinal lymph node staging is essential for optimal treatment decisions in patients with non-small cell lung cancer (NSCLC) who do not have distant metastases.^1^,^2^ Current NSCLC guidelines recommend endosonographically guided needle biopsy of mediastinal lymph nodes as a reliable first-choice method, reducing the need for more invasive surgical staging.^1^ A combination of endobronchial ultrasound-guided transbronchial needle aspiration (EBUS-TBNA) and endoscopic ultrasound-guided fine-needle aspiration (EUSFNA) can define the mediastinal lymph node stage with a sensitivity of 91% and a negative predictive value (NPV) of 96%.^1^ Mediastinal lymph nodes can also be non-invasively characterised by conventional brightness mode (B-mode) EBUS.^2^,^3^ Power/colour Doppler-mode image analysis of vascular patterns of lymph nodes can be helpful in predicting metastatic infiltration during the EBUS-TBNA procedure.^4^

Ultrasound elastography is an imaging procedure that can assess the biomechanical characteristics of different tissues and their deformation under compression.^5^ Malignant tissues are generally stiffer than native healthy tissues and can therefore be distinguished on the basis of decreased elasticity. Elastographic image analysis is based on qualitative pattern analysis and/or on semi-quantitative histogram analysis. New EBUS software supports visualisation of the tissue elasticity modulus by colour-coding tissue deformability: the hardest tissues are shown in blue, intermediate tissues in green and soft tissues in red. Moreover, it is possible to quantify the strain in two operator-selected areas. Comparing these different areas of tissue allows a numeric representation of the strain ratio between the two areas.

Ultrasound elastography has previously been applied as an external procedure (for example, in the diagnosis of thyroid, breast and prostate tumours) and as an endoscopic procedure (for example, in the diagnosis of pancreatic tumours, and nodal involvement of rectal and oesophageal cancer).^6^–^13^ A meta-analysis of EUS elastography trials on the differentiation of benign and malignant lymph nodes reported a sensitivity of 88%, a specificity of 85% and an area under the receiver operating characteristic (ROC) curve of 0.95.^14^ However, only one of the included trials evaluated the strain ratio as a diagnostic standard.^15^ A recent preliminary report on endobronchial ultrasound elastography suggests that this method may improve diagnostic yield.^16^

The aim of this pilot study was to define for the first time the diagnostic value of the EBUS elastography strain ratio for mediastinal lymph node staging in patients who were suspected to have lung cancer. Diagnostic values for the strain ratios were compared with EBUS B-mode features of selected mediastinal lymph nodes and with cytological diagnosis.

## Patients and methods

### Patients

This prospective single-centre study was conducted between August and December 2013. Evaluations were performed on consenting consecutive patients who were at least 18 years old and been referred for bronchoscopy with a suspicion of lung cancer according to a chest CT scan. Eligible patients had enlarged but discrete N2/N3 lymph nodes, a centrally located tumour with normal-sized mediastinal lymph nodes, or enlarged N1 lymph nodes with normal-sized mediastinal lymph nodes. Exclusion criteria were metastatic disease, severe co-morbidity that disqualified surgical treatment, mediastinal tumour infiltration, and small peripheral lung tumours with normalsized mediastinal lymph nodes.

Written informed consent was obtained from each patient prior to bronchoscopy. The study was approved by the National Medical Ethics Committee and was registered at ClinicalTrials. gov under the clinical trial number NCT02009319.

### Instruments and procedure

All bronchoscopy procedures were performed under deep sedation that was carried out by an anaesthesiologist. EBUS procedures were performed with two BF-UC180F linear ultrasound bronchoscopes (Olympus Tokyo, Japan). Real-time EBUS B-mode and elastography with strain ratio measurements were performed using a preproduction model of the Endoscopic Ultrasound Center EU-Y0008 (Olympus Tokyo, Japan), which was later commercialised under the name EU-ME2 Premier Plus after minor modifications.

Mediastinal lymph nodes were initially evaluated by B-mode EBUS as in Fujiwara *et al*. for anatomical location, size, shape, border distinction, echogenicity, central hilar structure and coagulation necrosis.^3^ A size of greater than 10 mm, round shape, distinct margin, heterogeneous echogenicity, absence of central echogenic hilum and coagulation necrosis were considered to be signs of malignant infiltration of the lymph node.

**FIGURE 1.**
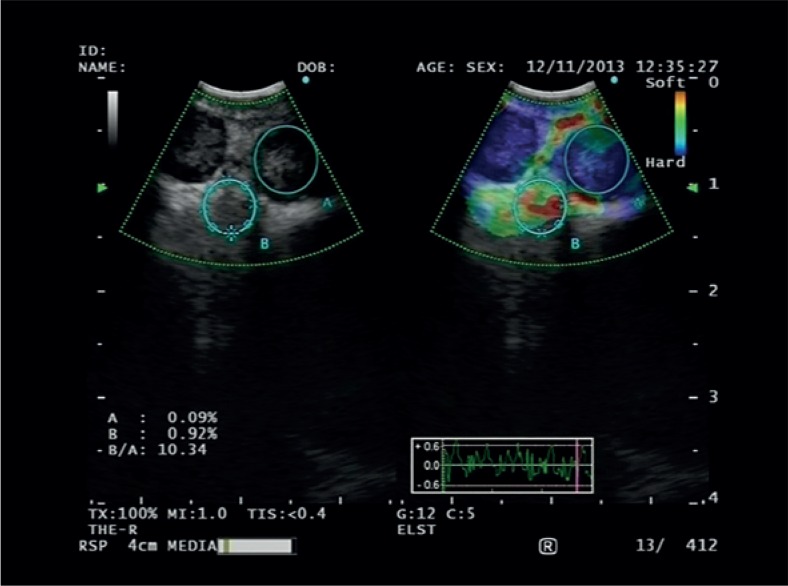
Endobronchial ultrasound (EBUS) elastography image of subcarinal lymph nodes. The image on the left-hand side displays greyscale ultrasound features. The image on the right-hand side is a superimposed elastographic image with a colour-coded scale (the hardest tissues are shown in blue and the softest in red). Symbol B/A represents the strain ratio, calculated between selected areas of the lymph node and the surrounding tissue.

The region of interest (ROI) for the elastographic evaluation was selected using a trackball, avoiding large vessels because structures with very low elasticity might induce artefacts in the evaluation of stiffness distribution.^13^ The elastography pattern as the result of tissue compression was produced by vascular pulsations and respiratory movement and not by direct bronchoscope pressure on the bronchial wall. After obtaining an artefact-free image, the “freeze” function was used, and the largest possible area of the lymph node was outlined to determine the strain. As a reference, an area of normal-appearing soft tissue from the surrounding mediastinum was selected to determine the strain ratio by EBUS processor unit (Figure 1). We selected tissue between the lymph node and bronchial cartilage or lateral to the lymph node. The strain ratio was measured twice for each selected lymph node. Whenever possible, the EBUS bronchoscope was inserted into the oesophagus to determine the strain ratio of the same lymph nodes to assess the influence of tracheal cartilage on the transtracheal measurements.

After non-invasive evaluation, EBUS-TBNA was performed using a 22-gauge needle. The pathologist who performed the cytological analysis was blinded to the strain ratio and other EBUS B-mode features.

### Definitive diagnosis

EBUS-TBNA was performed at least twice per lymph node. Malignancy, where present, was accepted as a definitive diagnosis. Patients with a benign outcome from EBUS-TBNA (*i.e*., lymphocytes) and proven cancer were sent for surgical treatment with lymph node dissection and histological examination. In cases where malignant disease was excluded, those patients were followed up meticulously until a benign outcome for the course of the disease was also confirmed.

### Data analysis

Data are presented as frequencies, ranges, means ± SD and percentages. The strain ratio for each lymph node was measured twice, and the mean of both measurements was accepted for further analysis. Sensitivity, specificity, accuracy, positive predictive values (PPVs), and NPVs were calculated. ROC analysis was performed to show the specificity/sensitivity for different strain ratio cut-off values. The area under the ROC curve was calculated. The optimal cut-off value for the strain ratio was selected at the point with the highest sensitivity and specificity. A paired *t*-test was used to analyse tracheal and oesophageal measurements of the strain ratio. The analysis was performed using GraphPad Prism version 5.00 (GraphPad Software, San Diego, California, USA; www.graphpad.com).

## Results

### Patients and lymph nodes

**FIGURE 2.**
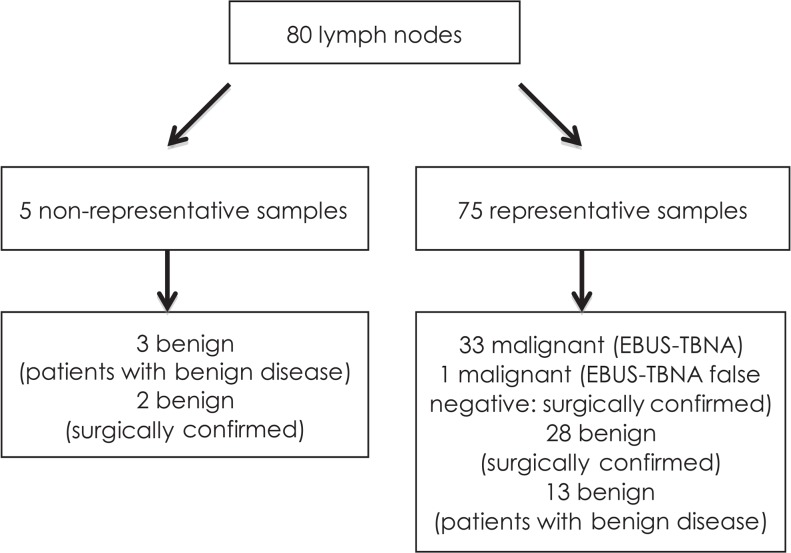
Flow diagram of lymph node confirmation. EBUS = endobronchial ultrasound

EBUS elastography was performed on 80 lymph nodes at different lymph node stations in 33 patients (25 male and 8 female) with an average age of 67.5 (± 8.2) years. Twenty-seven patients had a final diagnosis of lung cancer (14 adenocarcinoma, 8 squamous cell carcinoma, 3 small cell carcinoma and 2 non-small cell carcinoma); six patients had benign diagnoses at the end of the study (2 post-tuberculotic changes, 2 pneumonia/lung abscesses, one sarcoidosis and 1 infectious mononucleosis). Cytological specimens were adequate from 75 (93.7%) lymph nodes and non-representative from five (6.3%) lymph nodes. Malignant infiltration was confirmed in 34 (42.5%) lymph nodes, primarily by EBUS-TBNA (sensitivity 97.1%, NPV 97.6%; Figure 2).

The average size of the evaluated lymph nodes was 11.1 (± 4.8) mm, with a range of 4–26 mm. Thirty-three (41.3%) lymph nodes were larger than 10 mm, and forty-seven (58.7%) were 10 mm or smaller at the shortest diameter.

### EBUS elastography strain ratio

**FIGURE 3.**
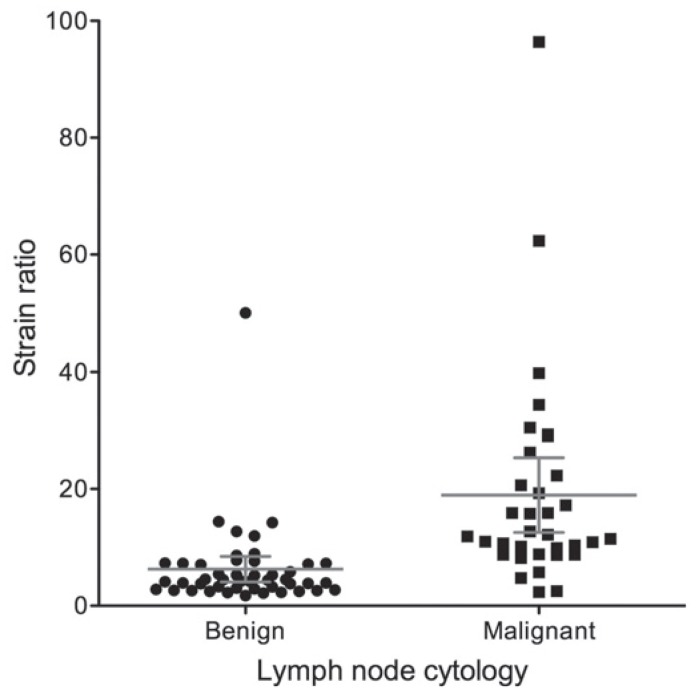
Plot of elastography strain ratio for benign and malignant lymph nodes at a cut-off of 8. Bars represent mean with 95% confidence interval (CI) of the mean.

**FIGURE 4.**
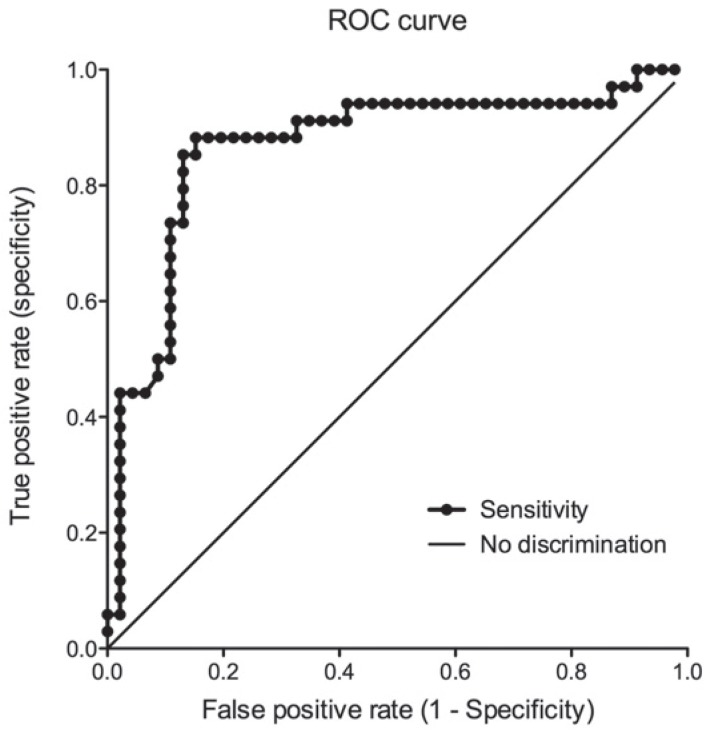
Receiver operating characteristic (ROC) curve for elastography strain ratio. Area under the ROC curve was 0.87 (*p* < 0.0001).

**FIGURE 5.**
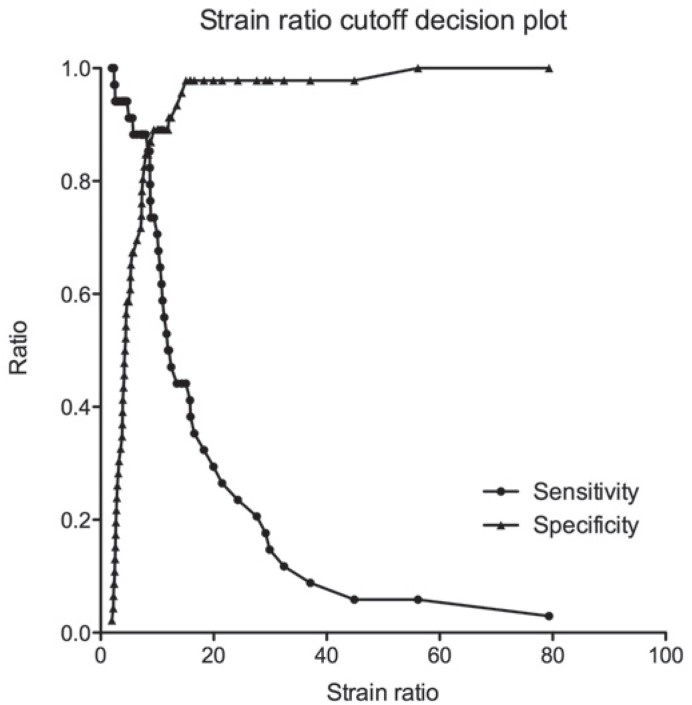
Sensitivity and specificity decision plot to determine the optimal cut-off for strain ratio. Curves cross at the strain ratio value 8.

The mean strain ratio for malignant lymph nodes was 18.96 ± 18.32 and 6.27 ± 7.30 for benign lymph nodes (Figure 3). The ROC area under the curve for the strain ratio was 0.87 (95% confidence interval [CI] 0.78–0.96, *p* < 0.0001; Figure 4). The optimal cut-off point for distinguishing malignant and benign lymph nodes, determined by a ROC sensitivity/specificity decision plot was at the strain ratio of 8 (Figure 5). Strain ratio values of 8 or higher represented the probability of malignant involvement with an accuracy of 86.25% (sensitivity 88.24%, specificity 84.78%, PPV 81.08%, NPV 90.70%; Table 1). There was no correlation between lymph node size and strain ratio when benign and malignant lymph nodes were evaluated separately.

**FIGURE 6.**
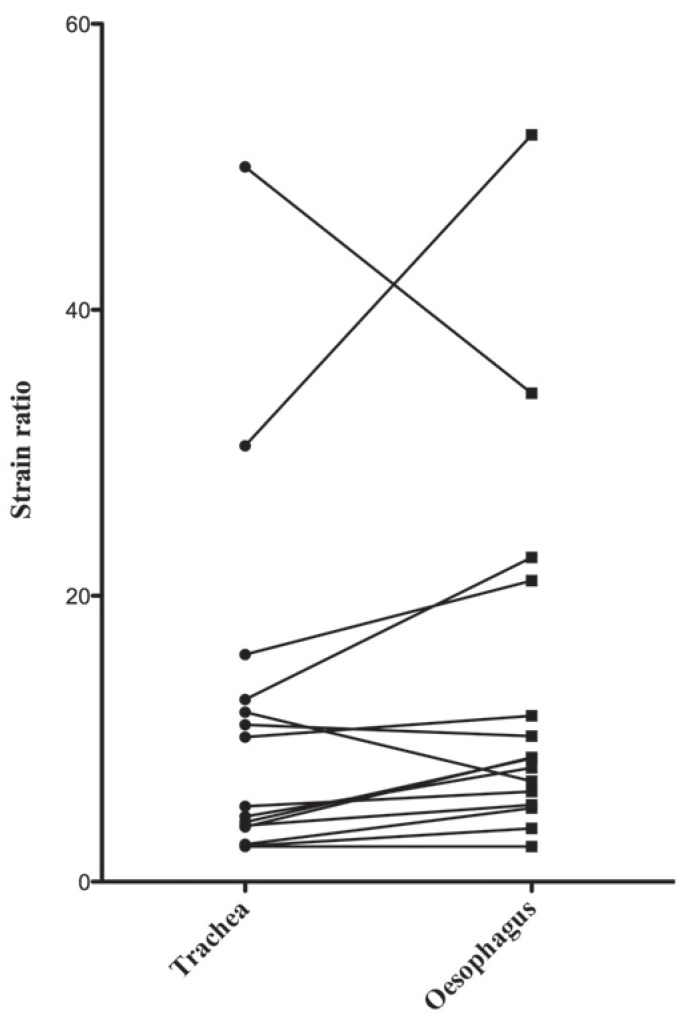
Strain ratios of the same mediastinal lymph nodes evaluated through tracheal and oesophageal approaches.

Fifteen lymph nodes at stations 7 and 4L were evaluated through tracheal and oesophageal approaches of an EBUS bronchoscope inserted consecutively (Figure 6). The method of strain ratio measurement was statistically insignificant (*p* = 0.26), although pairs of strain ratio measurements correlated significantly (*p* < 0.0001).

### Elastography strain ratio vs. EBUS B-mode features

The ability to differentiate between malignant and benign lymph nodes based on the elastography strain ratio was compared with the EBUS B-mode features of the same lymph nodes; the data are shown in Table 1. Elastography strain-ratio assessment was more accurate than any other lymph node characteristic assessed using EBUS B-mode criteria.

### Subgroup analysis

Subsequently, a group of patients with malignant disease (lung cancer) was analysed separately. Again, the optimal cut-off point for distinguishing malignant and benign lymph nodes was at the strain ratio of 8. The ROC area under the curve for the strain ratio was 0.91 (95% CI 0.83–0.98, *p* < 0.0001). Strain ratio values higher than 8 represented a probability of malignant involvement with an accuracy of 89.39% (sensitivity 88.24%, specificity 90.61%, PPV 90.91%, NPV 87.88%).

Eleven (28.21%) of 39 normal-sized lymph nodes sampled in patients with malignant disease were metastatic. The statistical data for the strain ratios in the group of normal-sized lymph nodes were as follows: acc. 89.74%, sensitivity 90.91%, specificity 89.29%, PPV 76.92%, and NPV 96.15%.

## Discussion

The strain ratio determined by endobronchial ultrasound elastography seems to be a promising new method for differentiating between malignant and benign lymph nodes in patients with lung cancer. This study confirmed the feasibility of elastography and strain ratio analysis using an EBUS bronchoscope in mediastinal lymph nodes. Moreover, the initial results showed a higher accuracy in the differentiation of malignant and benign lymph nodes than the conventional non-invasive EBUS modalities evaluated in this study.

The most accurate methods currently available for mediastinal staging in NSCLC patients are FNA under ultrasonic guidance and mediastinoscopy.^1^ These methods require tissue sampling and disrupt the integrity of mediastinal structures, carrying the risk of complications such as bleeding, infection and pneumothorax.^17^ Although rapid on-site evaluation (ROSE) can reduce the number of punctures, many patients are nonetheless exposed to numerous sampling procedures at several different lymph node stations and to subsequent mediastinoscopy.^18^,^19^ Less invasive techniques for accurate mediastinal lymph node diagnosis are therefore desirable for optimal mediastinal staging.

Cervical mediastinoscopy, which was the golden standard for mediastinal staging, seems to have limited utility in the patients with T1 and T2 clinically staged N0 by positron emission tomography - computed tomography (PET-CT).^20^ However, in case of CT-enlarged or PET-positive mediastinal lymph nodes, tissue confirmation is indicated.^2^ Endosonography (EBUS/EUS) with fine needle aspiration is still the first choice since it is minimally invasive and has a high sensitivity to rule in mediastinal nodal disease. If negative, surgical staging with nodal dissection or biopsy is indicated. Video-assisted mediastinoscopy is preferred over mediastinoscopy.^21^

The elastography strain ratio as a semi-quantitative method has been evaluated as part of an endoscopic procedure in gastroenterology, and favourable results have been reported for mediastinal lymph node analysis in patients with oesophageal cancer.^15^ The conclusions from the staging of upper gastrointestinal cancer have been less promising.^22^ Elastography without a strain ratio determination has been shown to be feasible during an EBUS procedure on a small group of patients.^16^

Our preliminary results describe for the first time the value of the strain ratio for the mediastinal staging of patients with lung cancer. A strain ratio cut-off point 8 had the highest ratio between sensitivity and specificity (sensitivity 88.24%, specificity 84.78%). However, the sensitivity and NPV of the strain ratio were still lower than for EBUS-TBNA.

In addition to 34 cytologically confirmed malignant lymph nodes, there were seven cases with false-positive elastography strain ratios. Three of these lymph nodes with prominent strain-ratio values contained substantial calcium deposits and were found in patients with post-tuberculotic lymph node abnormalities.

In four cases, the elastography strain ratio gave a false-negative result. Two lymph nodes were small and only partially overgrown with malignant tissue, which was identified upon subsequent histological examination. One of the lymph nodes was also a false negative on EBUS-TBNA. The other two lymph nodes were larger, heterogeneous and necrotic on conventional EBUS. Possible explanations for the false-negative results may lie in the highly vascularised or necrotic structure of particular nodes that appeared soft under elastographic evaluation.

In the subgroup of normal-sized mediastinal lymph nodes in patients with lung cancer, we found that 28.21% of the lymph nodes had malignant infiltration. The interesting feature was the high NPV (96.15%) of the strain ratio for this subgroup of lymph nodes, which was comparable to the EBUS-TBNA NPV. The potential role of the strain ratio may be in excluding lymph nodes with benign features from further invasive sampling. That would result in reduced invasiveness and reduced costs for mediastinal staging.

In general, EBUS elastography cannot currently replace the more accurate EBUS-FNA for mediastinal lymph node diagnosis. However, it may be useful as a supplemental method to reduce the number of punctures.^14^ This method can help the operator to select stiffer sections of lymph nodes for FNA to avoid necrosis or blood vessels, thus improving the quality of the samples. It could also help in choosing the most suspicious lymph node for sampling in regions where multiple lymph nodes are present. Some punctures could possibly even be avoided in groups of normal-sized lymph nodes that have a low strain ratio.

Several technical challenges were encountered during the study. Elastography relies on the movement and compression deformation of observed tissues. Obtaining a good and consistent recording was more problematic in the right paratracheal region, particularly in certain patients with more mediastinal fat tissue. Our impression was that heart and vascular pulsations were less pronounced on the right side of the trachea, and thus less tissue displacement could impede strain calculation.

In most cases, tracheobronchial cartilage did not interfere with image acquisition, and the strain ratios of the same lymph nodes measured through the trachea and through the oesophagus showed a strong correlation. However, in several patients, calcified cartilage cast intense acoustic shadows and narrowed the field of vision.

The third challenge was a scarceness of tissue in the mediastinum and the presence of large vessels. Although we tried to avoid the inclusion of large vessels in the ROI, this was not entirely possible in all cases.

The study design had several limitations. The first limitation was that this was a single-centre, single-operator scheme, and thus no inter-observer variations were taken into account. The gold standard for the determination of malignant involvement is a combination of several methods: a positive EBUS-TBNA result (assuming that there were no false-positive results), lymph node dissection during surgery (with station evaluation rather than direct node-to-node comparison) and a final benign diagnosis in the remaining patients. Because the strain ratio was determined from “frozen” EBUS images, selection bias might be created during static image selection, especially during the selection of the reference area in the strain ratio evaluation.

In conclusion, this study shows that EBUS-guided elastography with strain ratio assessment can distinguish malignant and benign mediastinal lymph nodes with greater accuracy than conventional EBUS modalities. The high NPV for normalsized mediastinal lymph nodes in lung cancer patients is comparable to the NPV obtained with EBUS-TBNA. This new method may potentially reduce the number of mediastinal EBUS-TBNAs and thus reduce the invasiveness and expense of mediastinal staging in NSCLC patients. Further multicentre trials are needed to confirm these preliminary results.

## Figures and Tables

**TABLE 1.** Sensitivity, specificity, positive predictive value (PPV), negative predictive value (NPV) and accuracy of each endobronchial ultrasound image category for metastatic lymph nodes in comparison to strain ratio (%)

	**Size (cm)**	**Shape**	**Margin**	**Echogenicity**	**CHS absent**	**CNS present**	**Strain ratio**
**Sensitivity**	67.65	76.47	79.41	67.65	88.24	41.18	88.24
**Specificity**	78.26	78.26	56.52	84.78	69.57	91.30	84.78
**PPV**	69.70	72.22	57.44	76.67	68.18	77.78	81.08
**NPV**	76.60	81.82	78.79	78.00	88.89	67.74	90.70
**Accuracy**	73.75	77.50	66.25	77.50	77.50	70.00	86.25

CHS = central hilar structure; CNS = coagulation necrosis sign

## References

[1] Silvestri GA, Gonzalez AV, Jantz MA, Margolis ML, Gould MK, Tanoue LT (2013). Methods for staging non-small cell lung cancer: Diagnosis and management of lung cancer, 3rd ed: American College of Chest Physicians evidence-based clinical practice guidelines. Chest.

[2] Cistaro A, Quartuccio N, Mojtahedi A, Fania P, Filosso PL, Campenni A (2013). Prediction of 2 years-survival in patients with stage I and II non-small cell lung cancer utilizing 18F-FDG PET/CT SUV quantification. Radiol Oncol.

[3] Fujiwara T, Yasufuku K, Nakajima T, Chiyo M, Yoshida S, Suzuki M (2010). The utility of sonographic features during endobronchial ultrasound-guided transbronchial needle aspiration for lymph node staging in patients with lung cancer: a standard endobronchial ultrasound image classification system. Chest.

[4] Nakajima T, Anayama T, Shingyoji M, Kimura H, Yoshino I, Yasufuku K (2012). Vascular image patterns of lymph nodes for the prediction of metastatic disease during EBUS-TBNA for mediastinal staging of lung cancer. J Thoracic Oncol.

[5] Saftoiu A, Vilman P (2006). Endoscopic ultrasound elastography - a new imaging technique for the visualization of tissue elasticity distribution. J Gastrointestin Liver Dis.

[6] Barr RG, Destounis S, Lackey LB, Svensson WE, Balleyguier C, Smith C (2012). Evaluation of breast lesions using sonographic elasticity imaging: a multi-center trial. J Ultrasound Med.

[7] Brock M, von Bodman C, Palisaar RJ, Löppenberg B, Sommerer F, Deix T (2012). The impact of real-time elastography guiding a systematic prostate biopsy to improve cancer detection rate: a prospective study of 353 patients. J Urol.

[8] Lyshchik A, Higashi T, Asato R, Tanaka S, Ito J, Mai JJ (2005). Thyroid gland tumor diagnosis at US elastography. Radiology.

[9] Giovannini M, Hookey LC, Bories E, Pesenti C, Monges G, Delpero JR (2006). Endoscopic ultrasound elastography: the first step towards virtual biopsy? Preliminary results in 49 patients. Endoscopy.

[10] Saftoiu A, Vilmann P, Hassan H, Gorunescu F (2006). Analysis of endoscopic ultrasound elastography used for characterisation and differentiation of benign and malignant lymph nodes. Ultraschall Med.

[11] Giovannini M, Thomas B, Erwan B, Christian P, Fabrice C, Benjamin E (2009). Endoscopic ultrasound elastography for evaluation of lymph nodes and pancreatic masses: a multicenter study. World J Gastroenterol.

[12] Janssen J, Dietrich CF, Will U, Greiner L (2007). Endosonographic elastography in the diagnosis of mediastinal lymph nodes. Endoscopy.

[13] Saftoiu A, Vilmann P, Ciurea T, Popescu GL, Iordache A, Hassan H (2007). Dynamic analysis of EUS used for the differentiation of benign and malignant lymph nodes. Gastrointest Endosc.

[14] Xu W, Shi J, Zeng X, Li X, Xie WF, Guo J (2011). EUS elastography for the differentiation of benign and malignant lymph nodes: a meta-analysis. Gastrointest Endosc.

[15] Paterson S, Duthie F, Stanley A (2012). Endoscopic ultrasound-guided elastography in the nodal staging of oesophageal cancer. World J Gastroenterol.

[16] Trosini-Desert V, Jeny F, Taillade L, Vignot S, Zribi H, Capron F (2013). Bronchial endoscopic ultrasound elastography: preliminary feasibility data. Eur Respir J.

[17] Asano F, Aoe M, Ohsaki Y, Okada Y, Sasada S, Sato S (2013). Complications associated with endobronchial ultrasound-guided transbronchial needle aspiration: a nationwide survey by the Japan Society for Respiratory Endoscopy. Respir Res.

[18] Lee HS, Lee GK, Lee HS, Kim MS, Lee JM, Kim HY (2008). Real-time endobronchial ultrasound-guided transbronchial needle aspiration in mediastinal staging of non-small cell lung cancer: how many aspirations per target lymph node station?. Chest.

[19] Trisolini R, Cancellieri A, Tinelli C, Paioli D, Scudeller L, Casadei GP, Parri (2011). Rapid on-site evaluation of transbronchial aspirates in the diagnosis of hilar and mediastinal adenopathy: a randomized trial. Chest.

[20] Fernandez FG, Kozower BD, Crabtree TD, Force SD, Lau C, Pickens A (2015). Utility of mediastinoscopy in clinical stage I lung cancers at risk for occult mediastinal nodal metastases. J Thorac Cardiovasc Surg.

[21] De Leyn P, Dooms C, Kuzdzal J, Lardinois D, Passlick B, Rami-Porta R (2014). Preoperative mediastinal lymph node staging for non-small cell lung cancer: 2014 update of the 2007 ESTS guidelines. Transl Lung Cancer Res.

[22] Larsen MH, Fristrup C, Hansen TP, Hovendal CP, Mortensen MB (2012). Endoscopic ultrasound, endoscopic sonoelastography, and strain ratio evaluation of lymph nodes with histology as gold standard. Endoscopy.

